# Climate and food diversity as drivers of mammal diversity in Inner Mongolia

**DOI:** 10.1002/ece3.4908

**Published:** 2019-01-16

**Authors:** Gang Feng, Hui Yan, Xueting Yang

**Affiliations:** ^1^ Ministry of Education Key Laboratory of Ecology and Resource Use of the Mongolian Plateau & Inner Mongolia Key Laboratory of Grassland Ecology, School of Ecology and Environment Inner Mongolia University Hohhot China; ^2^ Monitoring and Planning Institution of Inner Mongolia Forestry Administration Hohhot China

**Keywords:** biotic interactions, current climate, environmental heterogeneity, food diversity, species area relationships, species richness

## Abstract

Traditionally, geographical distribution of biodiversity is assumed to be codetermined by multiple factors, for example, temperature, precipitation, environmental heterogeneity, and biotic interactions. However, few studies have simultaneously compared the relative roles of these factors in shaping the mammal diversity patterns for different feeding groups, that is, herbivores, insectivores, and carnivores. In this study, we assessed the relations between mammal diversity and current climate (mean annual temperature and precipitation), altitudinal range as well as mammal's food diversity in Inner Mongolia. Our results showed that the species richness for the three feeding guilds of mammals consistently increased with their food diversity, that is, species richness of plants, insects, and rodents. Mammal diversity also significantly decreased with mean annual temperature and precipitation. Random Forest models indicated that climate and food diversity were always included in the combinations of variables most associated with mammal diversity. Our findings suggest that while climate is an important predictor of large scale distribution of mammal diversity, biotic interactions, that is, food diversity, could also play important roles.

## 1 INTRODUCTION

Biodiversity distribution at macro‐ecological scale and its multiple drivers is a key question in ecology and biogeography (Fine, 2015; Pärtel, Bennett, & Zobel, 2016; Staniczenko, Sivasubramaniam, Suttle, & Pearson, 2017). Regional and historical factors, for example, current climate and paleoclimate change, have been widely linked with geographical distribution of mammal diversity (Samuels & Hopkins, 2017; Svenning, Fløjgaard, & Baselga, 2011). Meanwhile, mammal diversity is also highly associated with other factors, for example, biotic interactions and environmental heterogeneity (Kissling & Schleuning, 2015; Stein et al., 2015). However, few studies have simultaneously assessed the relative roles of these factors in shaping mammal distribution at large scales, especially for different feeding groups.

Specifically, current climate could shape biodiversity distribution patterns directly by setting limits of species physiological tolerances, and indirectly by limiting the energy availability (Rowe, 2009). Environmental heterogeneity would affect species richness by providing more available niche space, more shelter and refuges from adverse environment, and higher probability of species diversification (Stein et al., 2015). Trophic interactions, for example, prey–predator and plant–herbivore, could influence community composition and structure at local and regional scales through a network including the dependent interacting species (Kissling & Schleuning, 2015).

Due to a large range of longitude (29° and 3,000 km from northeast to southwest), Inner Mongolia has a clear gradient of climate, for example, from northeast to southwest mean annual precipitation (MAP) decreases from 450 to 40 mm, and mean annual temperature (MAT) increases from −2 to 6°C. Therefore, Inner Mongolia also has diverse vegetation types (forest, grassland, and desert) and high biodiversity (2,447 vascular plant species, 467 bird species, and 149 mammal species) (Xu, 2007, 2015, 2016a, 2016b; Zhao, 2012),making it an ideal place to study the geographical distribution of mammal diversity. However, no study has quantitatively assessed the patterns and drivers of geographical distribution of mammal diversity in this region.

In this study, we first divided all mammal species into three groups according to their food resources, for example, herbivores, insectivores, and carnivores (mainly rodent predators). We then assessed the association between food diversity, altitudinal range, current climate, and diversity of the three groups of mammals, respectively. Because the distribution data of mammals are at county level and area of each county varies widely (100–90,000 km^2^), we also assessed the relations between county area and mammal diversity. In all, we assumed that food diversity would be positively associated with mammal diversity through the network connecting these interacting species and would codetermine the geographical distribution of mammal diversity with current climate and altitudinal range.

## 2 MATERIALS AND METHODS

Distribution data of mammals at county scale (86 counties) were compiled from the fifth and sixth volumes of Fauna of Inner Mongolia (Xu, 2016a, 2016b). Information about food resources of each mammal was also from Fauna of Inner Mongolia (Xu, 2016a, 2016b). We used a general definition of herbivores, insectivores, and carnivores (mainly feed on rodent), for example, an omnivore was placed into all the three groups, and if an animal feeds on both insects and plants, it was labeled as both insectivore and herbivores. Therefore, we finally had 107 herbivore species, 81 insectivore species, and 41 carnivore species (Supporting Information Appendix S1). Distribution data of plants and insects at county scale were obtained from Chinese Vascular Plant Distribution Database (compiled from Flora Reipublicae Popularis Sinnicae) and Insects of Inner Mongolia China (Delecti Florae Reipublicae Popularis Sinicae Agendae Academiae Sinicae ed, [Link]–2004; Neng, 1999). Current climate was represented by MAT, MAP. MAT and MAP were collected from Worldclim, which represented the mean value between 1960 and 1990 (Hijmans, Cameron, Parra, Jones, & Jarvis, 2005). We calculated the mean values for each county using the county‐level shapefile. Altitudinal range (the maximum value minus the minimum value of each county) was computed based on a digital elevation model available in the same source and was used as a proxy of environmental heterogeneity (Feng et al., 2016; Stein, Gerstner, & Kreft, 2014).The cell size of MAT, MAP, and altitude is 2.5 min. The County area was obtained from the county‐level shapefile map of Inner Mongolia.

To analyze the univariate relationships between potential drivers and mammal diversity, ordinary least squares (OLS) models were used. Species richness of herbivores, insectivores, carnivores, plants, insects and rodents, as well as county area were log transformed to get normal distributed residuals. All dependent and independent variables were standardized (mean = 0 and *SD* = 1) to make the regression coefficients comparable. To account the spatial autocorrelation of the regression residuals, simultaneous autoregressive (SAR) models were also used for the single variable analyses. Moran'I test was used to assess the spatial autocorrelation in the residuals.

The combinations of variables most associated with mammal diversity were assessed using Random Forest models, because it could deal with nonlinear relationships and interactions among variables, and does not require the data to follow strict assumptions, for example, normality in errors and homoscedasticity (Breiman, 2001). For each combination of variables, we ran the Random Forest models 1,000 times to randomly split the data (50% training data and 50% evaluation data), and the median of the 1,000 Pearson correlations between the predicted and observed mammal diversity was used to rank the combinations.

Since species richness of plant, insect, and rodent could also be affected by climate, altitudinal range, county area, we used StructuralEquation Models (SEM) to test the direct and indirect effects of these variables on mammal diversity. All analysis were conducted in R (R Development Core Team, 2016) using vegan (Oksanen et al., 2015), spdep (Bivand et al., 2015), randomForest (Liaw & Wiener, 2002), and lavaan (Rosseel, 2012) packages.

## 3 RESULTS

**Figure 1:**
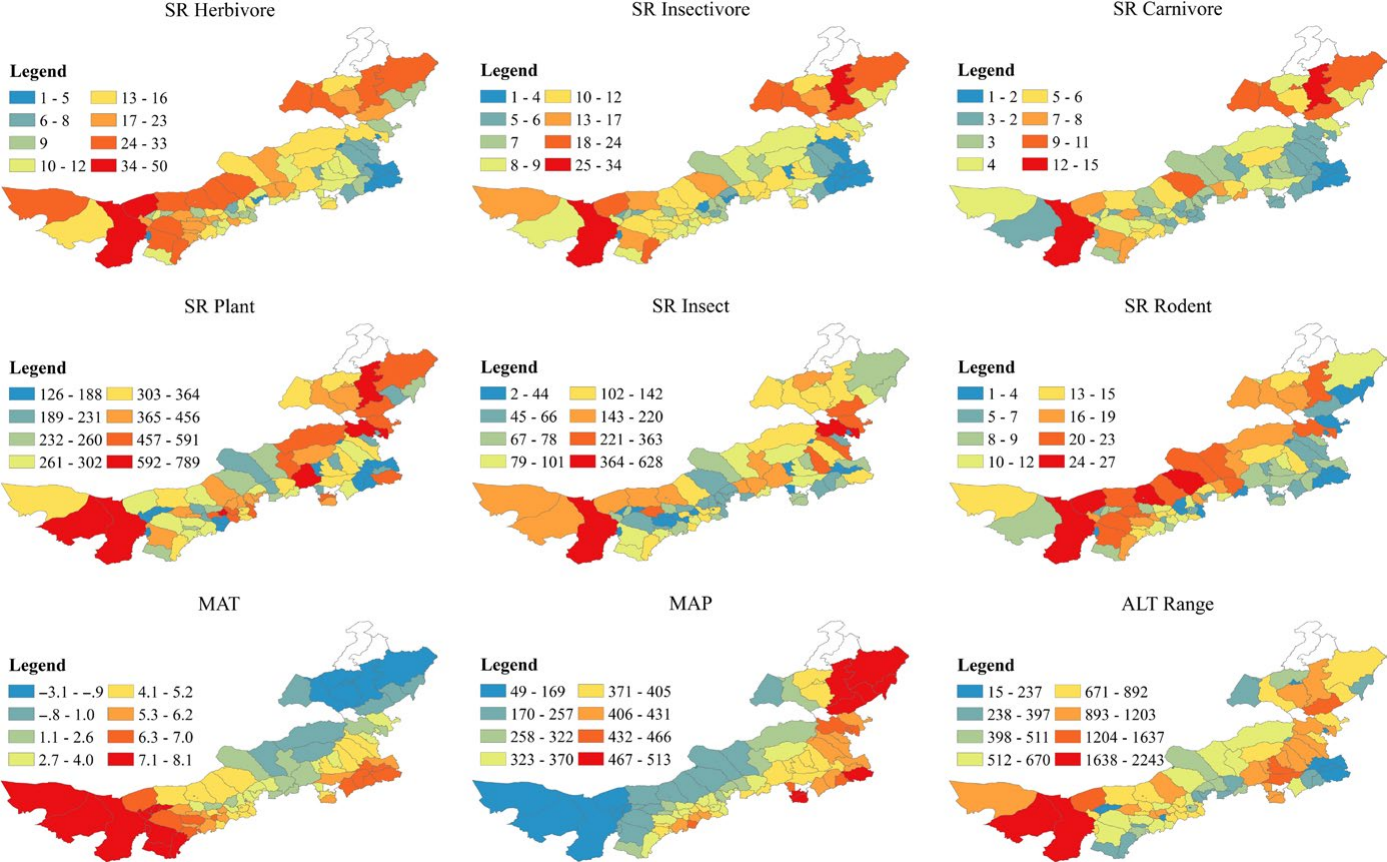
Maps of species richness for herbivores, insectivores, carnivores, plants, insects, rodents, mean annual temperature (MAT), mean annual precipitation (MAP), altitudinal range (ALT Range). The white regions are counties without species distribution information

The range of species richness for herbivores, insectivores, and carnivores among counties were one to 50, one to 34, and one to 15, respectively (Figure 1). The county with highest species richness for these three feeding groups was Alashanzuoqi (Western Inner Mongolia), which also tended to have highest food diversity, largest altitudinal range, highest temperature, and least precipitation (Figure 1).

**Figure 2:**
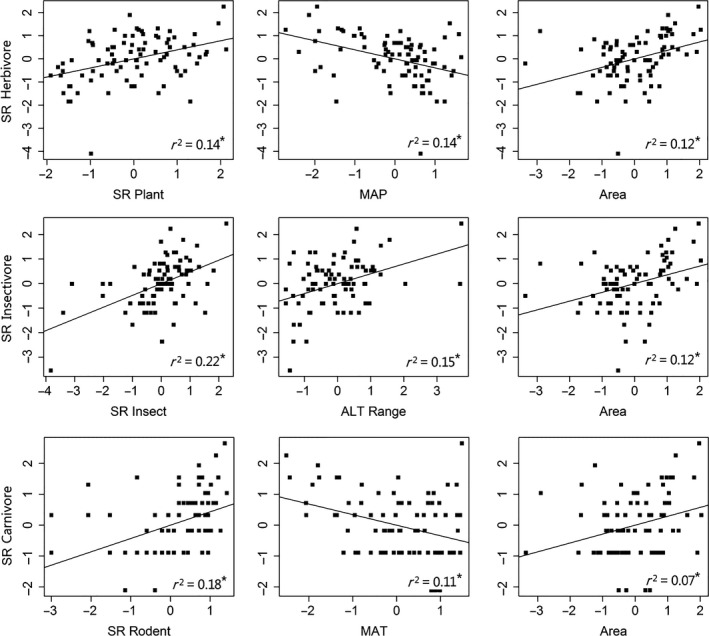
Scatter plot of species richness of herbivores, insectivores, carnivores, and their three most associated variables. Mammal diversity is positively correlated with food diversity and county area. ^*^ *p* < 0.01

**Figure 3:**
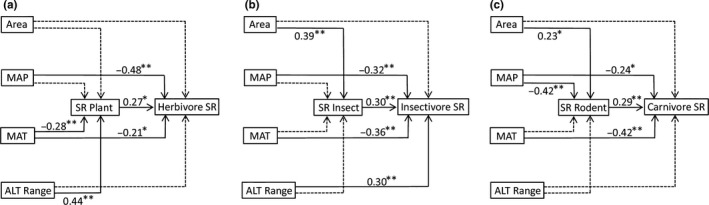
Results of structural equation models examining the direct and indirect effects of county area (Area), mean annual precipitation (MAP), mean annual temperature (MAT), altitudinal range (ALT Range), and food diversity (species richness of plant, insect and rodent) on mammal (herbivore [a]; insectivore [b]; carnivore [c]) diversity. Significant standardized regression coefficients were given. ^*^ *p* < 0.05, ^**^ *p* < 0.01

The single variable OLS models and structural equation models showed that species richness of herbivores, insectivores, and carnivores significantly increased with their food diversity, that is, species richness of plants, insects, and rodents, and significantly decreased with MAT and MAP (Figures 2 and 3; Table 1). County area was also positively correlated with mammal diversity, with species richness of carnivores least correlated (Figure 2; Table 1). SAR models showed similar patterns (Table 1).

**Table 1:** Single variable analyses by ordinary least squares (OLS) and simultaneous autoregressive (SAR) models

	Herbivore SR				Insectivore SR				Carnivore SR			
	Coef_ols_	*r* ^2^_ols_	Coef_SAR_	*R* ^2^_SAR_	Coef_ols_	*r* ^2^_ols_	Coef_SAR_	*r* ^2^_SAR_	Coef_ols_	*r* ^2^_ols_	Coef_SAR_	*r* ^2^_SAR_
SR Food	0.39	0.14**	0.37	0.40**	0.48	0.22**	0.40	0.57**	0.43	0.18**	0.35	0.43**
ALT Range	0.35	0.11**	0.39	0.41**	0.40	0.15**	0.39	0.53**	0.18	0.02	0.20	0.37*
MAT	−0.13	0	−0.14	0.26	−0.29	0.07**	−0.29	0.39	−0.34	0.11**	−0.25	0.34
MAP	−0.39	0.14**	−0.40	0.31**	−0.22	0.04*	−0.21	0.39	−0.21	0.03*	−0.27	0.35
Area	0.37	0.12**	0.39	0.41**	0.36	0.12**	0.33	0.49**	0.29	0.07**	0.20	0.37*

Notes

SR Food is species richness of plants, insects, and rodents for Herbivore SR, Insectivore SR, and Carnivore SR, respectively. MAT and MAP are mean annual temperature and precipitation. ALT_Range_ and area are the altitudinal range and area of each county. Coefficients and *r* ^2 ^were given.

* *p* < 0.05

** *p* < 0.01.

Random forest models showed that food diversity was always included in the six best combinations of variables for herbivore, carnivore, and insectivore (occurred in four of the six best combinations) (Table 2). MAT was included in the six best combinations of variables for carnivore and insectivore (Table 2). MAP was included in the six best combinations of variables for herbivore (Table 2).

**Table 2:** Results of random forest models showing the six combinations of variables most associated with mammal (herbivore, insectivore, and carnivore) diversity, ranked by the correlations between predicted and observed mammal diversity

	SR Food	MAT	MAP	ALT_Range_	Area	Cor_RF_
Herbivore						0.636
						0.635
						0.619
						0.610
						0.606
						0.588
Insectivore						0.570
						0.556
						0.549
						0.541
						0.540
						0.528
Carnivore						0.587
						0.565
						0.552
						0.538
						0.524
						0.510

Notes

Each column represented a variable: ALT_Range_: altitudinal range of each county; rea: area of each county; MAP: mean annual precipitation; MAT: mean annual temperature; SR Food: species richness of food items.

Black cells indicated that the variable was included in the particular combination (each row).

## 4 DISCUSSION

Our study is the first attempt to assess the patterns and multiple drivers of geographical distribution of mammal diversity focusing in Inner Mongolia, a region with diverse vegetation types and high biodiversity. We found that mammal diversity significantly decreased with MAP and MAT, and significantly increased with their food diversity. The best combination of variables predicting mammal diversity distribution patterns always include food diversity and current climate.

### 4.1 Biotic interactions and geographical distribution of mammal diversity

While it is a tenet that climate would shape the geographical distribution of biodiversity, an increasing number of studies have showed that biotic interactions also play important roles in limiting species distribution (Araújo & Rozenfeld, 2014; Kissling & Schleuning, 2015; Wisz et al., 2013).For example, global mammal predator richness is more associated with prey species richness than productivity, climate, and human influence (Sandom et al., 2013). Herbivore diversity in the Arctic is positively related with species richness of predators (Barrio et al., 2016). Consistent with these studies, our results also showed positive relations between herbivore diversity, insectivore diversity, carnivore diversity, and their food diversity, that is, species richness of plants, insects, and rodents, respectively. In addition, these relations are stronger than current climate and altitudinal range, providing strong supplementing evidence for the previous studies.

### 4.2 County area and geographical distribution of mammal diversity

Being a canonical law in ecology and biogeography, species–area relationship (SAR) is widely linked with species dispersal ability (Aranda et al., 2013; Rosenzweig, 1995). For example, the slope of SARs for spermatophytes (lower dispersal ability) is higher than for pteridophytes and bryophytes (higher dispersal ability) (Patiño et al., 2014). A study on grassland beetle diversity found that species with low dispersal ability always occur on large single sites (Noordwijk et al., 2015). In this study, species richness of herbivores and insectivores was more associated with county area than carnivores. Combining with a recent study on bird diversity also in Inner Mongolia, which found no relations between county area and bird species richness(Liang et al., 2018), our results supporting the findings of previous studies, that is, the higher dispersal ability of organisms, the weaker relations between their species richness and area. A main explanation for SAR is that larger regions tend to have higher environmental heterogeneity (Báldi, 2008). Supporting this explanation, we found that the county area is positively correlated with altitudinal range (*r* = 0.52, *p* < 0.01).

### 4.3 Current climate and geographical distribution of mammal diversity

Positive relations between mammal diversity and current climate, that is, temperature and precipitation, have been widely reported (Davies, Buckley, Grenyer, & Gittleman, 2011; Hawkins & Porter, 2003). However, there is also evidence against these positives relations, especially in arid and semi‐arid regions (Abramsky & Rosenzweig, 1984; Brown & Ernest, 2002; Ferrer‐Castán, Morales‐Barbero, & Vetaas, 2016). A study on desert rodents in Israel shows that rodent species richness first increases and then decreases with more rainfall, peaking in relatively dry locations (Abramsky & Rosenzweig, 1984). Mammal richness decreases with increasing energy because of limited water resources (Ferrer‐Castán et al., 2016). Consistent with these studies, our results also showed negative relations between mammal diversity and current climate, that is, MAP and temperature.

### 4.4 Altitudinal range and geographical distribution of mammal diversity

High environmental heterogeneity could support high species richness by providing more available niches, refuges, and probability of species diversification (Stein et al., 2015). Being a synthetic and easily quantified proxy for environmental heterogeneity, altitudinal range has been widely associated with mammal diversity (Qian, Badgley, & Fox, 2009; Stein et al., 2014). Supporting these studies, we also find a positive relation between altitudinal range and herbivore and insectivore diversity.

## 5 CONCLUSIONS

Being the first study assessing the patterns and multiple drivers of mammal diversity in Inner Mongolia, our results indicated that biotic interactions, that is, food diversity, was also an important factor. In addition, the mammal diversity was most associated with a combination of variables including food diversity and current climate, emphasizing the important roles of multiple factors in shaping geographical distribution of mammal diversity.

## CONFLICT OF INTEREST

None declared.

## AUTHOR CONTRIBUTIONS

HY collected the data and drafted the article. XY revised the article. GF designed the study, analyzed the data, drafted and revised the article.

## Data Availability

Data of species richness for different organism groups and other explanatory variables are available with the request to the corresponding author.
